# Sphenoid sinus mucocele masquerading as retrobulbar optic neuritis: a case report

**Published:** 2018

**Authors:** Abri Aghdam Kaveh, Ghazi-Zadeh Ahsaei Mahya, Zand Amin, Soltan Sanjari Mostafa

**Affiliations:** * Eye Research Center, Rassoul Akram Hospital, Iran University of Medical Sciences, Tehran, Iran

**Keywords:** optic neuritis, mucocele, sphenoid sinus

## Abstract

**Objective:** To describe an infrequent instance of sphenoid sinus mucocele presenting as retrobulbar optic neuritis and highlight the value of imaging in the diagnosis and treatment plans.

**Methods:** A woman aged 26 noted a sub-acute decrease in vision in the right eye, which mimicked optic neuritis. Magnetic resonance imaging (MRI) indicated a large mass in the sphenoid sinus, which was in favor of mucocele.

**Results:** Endoscopic sphenoidotomy and marsupialization of the mucocele were carried out, and the diagnosis was confirmed by pathology. The patient was also administered a high dose of corticosteroid, upon which progression of the disease was halted. Unfortunately, no significant improvement in vision was achieved.

**Conclusions:** This report emphasized the important role of imaging in differentiating between different causes of optic neuropathies. A high level of clinical skepticism along with appropriate imaging studies can help diagnose rare causes. With timely management, gratifying results may be achieved.

## Introduction

Sphenoid sinus mucoceles occur infrequently and have a prevalence of one percent of all paranasal sinus mucoceles [**1**]. They are frequently misdiagnosed and can result in complications because of their vicinity with important structures. Their most common presentation is headaches (frontal or retro-orbital: 70% of the patients) and the second common is visual disturbance (65%) [**2**]. Further complications are ocular palsy (especially third and sixth nerve palsy), exophthalmos, and pituitary gland dysfunctions in rare cases. We describe an unusual incident of sphenoid sinus mucocele masquerading retrobulbar optic neuritis.

## Materials and Methods - Case Report

**Fig. 1:**
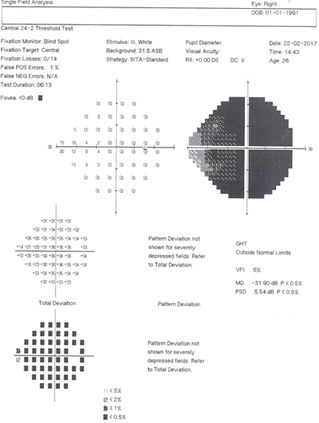
Humphrey’s visual field, SITA-standard protocol of patient’s right eye. Severe generalized depression in the right eye can be discerned

A 26-year-old female presented with a 3-weeks history of gradually advancing loss of the right eyesight, accompanied by irritating frontal headaches and painful eye movements. Her medical history was significant for asthma and nasal polyp. Prior to her referral, the patient had been treated by an otolaryngologist for pan-sinusitis with oral tablets of Co-amoxiclav (625mg/ three times per day for two weeks). Office examination revealed a best-corrected visual acuity of count fingers at forty centimeters in the right eye and 10/ 10 in the left eye (by Snellen E letter chart, with a distance of six meters). Her pupillary reactions were normal in both eyes with a 3+ relative afferent defect in the right eye. Although painful in the right eye, extraocular motilities were intact in both eyes. Intraocular pressures were within normal limits in both eyes by applanation tonometry. Color plate testing results (by Ishihara’s color plate test) for the right eye was not applicable, and for the left eye, it was 14/ 14. Slit-lamp examination was inconspicuous. Her dilated fundus exam of both eyes was completely normal including normal optic disc appearance and macula. Humphrey’s visual fields test of the right eye revealed a generalized depression defect (**Fig. 1**).

**Fig. 2:**
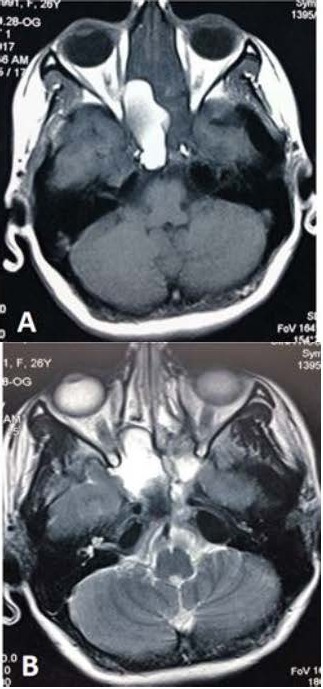
T1 (A) and T2 (B) weighted axial MRI shows a homogenous hyper-intense mass in the right sphenoid sinus, with invasion to the right optic canal and right posterior ethmoid sinus

The patient was admitted with the clinical impression of retrobulbar optic neuritis. Meanwhile, brain magnetic resonance imaging (MRI) with and without contrast was performed, which showed a homogenous cystic lesion that was compatible with mucocele in the right sphenoid sinus that extended into the sinus walls, right posterior ethmoid sinus and extra-orbital segment of the right optic nerve (**Fig. 2AB**). 

**Fig. 3:**
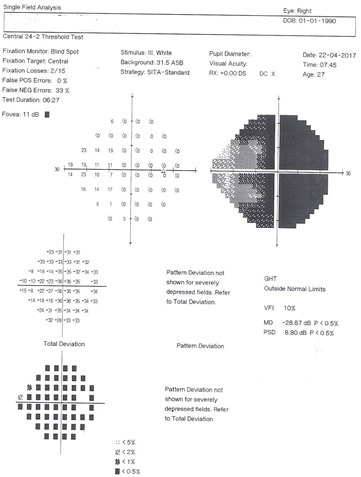
Postoperative visual field of the right eye. Slight improvement in the visual filed is obvious

Consequently, emergent otolaryngology consultation was requested. Simultaneously, 250 mg methylprednisolone was administered intravenously at every 6 hours. Otolaryngologists first performed an endoscopic polypectomy but were unable to remove the mucocele completely. They then partially resected the right turbinate, opened the fibrosis in the maxillary opening, and accessed the right sphenoid sinus that was filled with debris and mucopurulent discharge. They irrigated the debris and reduced the size of the mucocele. One week after removing the mucocele, the right eyesight recuperated to counting fingers at 1 meter and the visual field was also slightly improved (**Fig. 3**). Despite several attempts, these visual field improvements were the best ones that could be obtained for the patient. One month later, her vision was counting fingers at 3 meters in the right eye and optic atrophy appeared.

## Discussions

Optic neuritis denotes inflammation, demyelination, or degeneration of the optic nerve. Multiple sclerosis is the most frequent cause of unilateral optic neuritis. Other causes include infectious diseases (like syphilis), sarcoidosis, systemic lupus erythematous, neuromyelitis optica, nutritional deficiency (like vitamin B12 deficiency), and posterior paranasal sinus mucoceles (as an unusual source) [**2**].

Paranasal sinus mucoceles result from an accumulation of secretions and debris in the sinus cavity attributable to the obstruction of sinus ostium. Sphenoid sinus mucocele is a rare condition that is often misdiagnosed and can have important complications due to its close vicinity to structures such as the brain, orbital cavity, cavernous sinus and their components and the optic nerve. These complications include headache, visual disturbance, and endocrine disturbances. Ophthalmic findings include visual acuity impairment and visual field defect due to optic neuropathy, third or six nerve palsies due to the involvement of the cavernous sinus and exophthalmos caused by involvement of the orbital cavity.

The function of paranasal sinus in the occurrence of optic neuritis is controversial. Sinusitis and mucoceles can cause optic neuritis by theoretically numerous ways. One is the direct spread of inflammation to the optic nerve via bony structures of sinus cavity neighboring with optic nerve dural sheath [**3**,**4**]. Also, a compressive effect of the posterior ethmoid or the sphenoid sinus’s mucocele on the optic nerve can cause optic neuropathy. In Sphenoid mucocele, the vision deficit may be due either to the accumulation effect of the enlarging mucocele resulting in a disruption in the blood stream of the optic nerve or to the involvement of the optic canal causing unilateral, gradually proceeding to loss of vision, which can result in optic degeneration [**4**-**6**]. Other potential causes of optic neuritis during sinus diseases are allergic reactions and autoimmune demyelination of the optic nerve as a result of infectious organisms [**4**]. 

Computed Tomography (CT) scan is necessary for evaluating the optic canal and the adjacent paranasal sinuses in patients with sinus diseases accompanied with visual disturbances. In CT-scans we can find signs of sinusitis, abscesses formation, mucoceles or any dehiscences or destructions in posterior sinus walls [**7**].

A bulky mucocele generates a typical radiographic feature of an engorged deformed sinus with a bony defect showing a protrusion into the adjoining structures. MRI image of the nose and paranasal sinuses is vital to corroborate the diagnosis of mucocele [**8**]. It presents as a cystic homogenous lesion in relation to the paranasal sinuses. MRI is essential to find the extent of the mucocele as well [**7**]. Orbital MRI of our patient indicated a homogenous cystic lesion in the right sphenoid sinus with invasion to the sinus walls and optic nerve, which was suggestive for mucocele.

Unfortunately, the visual acuity and visual field defects rarely improve after mucocele surgical removal and only one fifth of the patients with visual impairment due to sphenoid sinus mucocele experience improvement of visual acuity to > 20/ 40 after surgery, during follow-up visits. But early surgical excision is necessary to limit the visual impairment complications [**9**]. In 2008, Pelaz et al. presented two cases of reversible optic neuritis due to sphenoid sinus disorders, which reverted in one case with surgical treatment and in the other with steroid-based treatment [**10**]. In 2012, Gupta et al. reported an instance of retrobulbar optic neuritis attributable to sphenoid sinus mucocele. In this case, early surgical excision of the mucocele brought about thorough recuperation from the lesion and improvement in contrast sensitivity and visual field [**11**]. In 2013, Sharifi et al. reported three instances of sudden blindness as a result of isolated sphenoid sinus mucocele and retention cyst. After the endoscopic removal of the mucoceles, total vision and visual field recovery in two cases and partial recovery in the third were achieved [**12**]. In 2014, Selvakumar et al. presented a rare instance of sphenoethmoidal mucocele leading to bilateral optic neuropathy and unilateral sixth nerve palsy. After the surgical excision of the mucocele, the vision was recovered and they observed thorough resolution of the sixth nerve palsy [**13**]. Any surgical approach should focus on reducing the size of mucocele to reduce the compressive effect of the lesion on the optic nerve. Treatment involves marsupialization or surgical excision of the mucocele, often resulting in rapid secession of some ophthalmic manifestations especially third nerve palsy, but with a smaller effect on visual impairments [**9**]. Regarding our patient, she had a small improvement in visual acuity throughout the follow-up period (from count fingers at forty centimeters to one meter) and mild visual field improvement in the right eye. However, after three months of her clinical presentation, optic atrophy arose in the affected eye, regardless of medical and surgical therapy of mucocele and optic neuritis.

There are few case reports regarding optic neuropathy due to sphenoid sinus mucoceles. Prompt diagnosis, referral, and treatment could recover the optic neuropathy and the vision may improve, but when the proper treatment is delayed, the prospects will not be promising. Neuroimaging studies ought to be supposed vital in cases of retrobulbar neuritis even in the incidence of a solitary typical feature.
